# Functionality of Beech Bark in Adhesive Mixtures Used in Plywood and Its Effect on the Stability Associated with Material Systems

**DOI:** 10.3390/ma12081298

**Published:** 2019-04-20

**Authors:** Roman Réh, Rastislav Igaz, Ľuboš Krišťák, Ivan Ružiak, Milada Gajtanska, Monika Božíková, Martin Kučerka

**Affiliations:** 1 Faculty of Wood Sciences and Technology, Technical University in Zvolen, 96001 Zvolen, Slovakia; reh@tuzvo.sk (R.R.); igaz@tuzvo.sk (R.I.); ruziak@tuzvo.sk (I.R.); gajtanska@tuzvo.sk (M.G.); 2 Faculty of Engineering, Slovak University of Agriculture in Nitra, 94976 Nitra, Slovakia; monika.bozikova@uniag.sk; 3 Faculty of Natural Sciences, Matej Bel University, 97401 Banská Bystrica, Slovakia; martin.kucerka@umb.sk

**Keywords:** beech bark, beech bark-based fillers, beech plywood, ecological fillers, urea formaldehyde adhesives, free formaldehyde

## Abstract

The results of research into utilizing grinded beech bark in order to substitute commonly used fillers in urea formaldehyde (UF) adhesive mixtures to bond plywood are presented in the present study. Four test groups of plywood with various adhesive mixtures were manufactured under laboratory conditions and used for experimentation. Plywood made using the same technology, with the common filler (technical flour), was used as a reference material. Three different concentrations of grinded beech bark were used. The thermal conductivity of the fillers used, viscosity and its time dependence, homogeneity and the dispersion performance of fillers were evaluated in the analysis of adhesive mixture. The time necessary for heating up the material during the pressing process was a further tested parameter. The produced plywood was analyzed in terms of its modulus of elasticity, bending strength, perpendicular tensile strength and free formaldehyde emissions. Following the research results, beech bark can be characterized as an ecologically friendly alternative to technical flour, shortening the time of pressing by up to 27%. At the same time, in terms of the statistics, the mechanical properties and stability of the material changed insignificantly, and the formaldehyde emissions reduced significantly, by up to 74%. The utilization of bark was in compliance with long-term sustainability, resulting in a decrease in the environmental impact of waste generated during the wood processing.

## 1. Introduction

Plywood production is an important issue, not only because of the importance of plywood among engineered wood products (EWPs), which are widely applied in residential construction, interior decoration and furniture industries, but also due to pressing environmental concerns [1,2]. In 2017, global wood-based panel production reached 402 million m^3^ [3], requiring 16.2 megatonnes of wood adhesives and binders [4,5]. 

Urea formaldehyde (UF), phenol formaldehyde, polyurethane (PU) and melamine urea formaldehyde (MUF) resins are the most common adhesive materials for the production of EWPs [6,7]. UF adhesives are the major resins and they are widely used in the wood industry because of their high bonding strength and lower cost when compared to other adhesives. On the other hand, using formaldehyde-based adhesives leads to the issue of formaldehyde emissions from the panels [8,9,10]. Formaldehyde is classified under carcinogenic category 1B and mutagen category 2 according to the Classification, Labelling and Packaging of Substances and Mixtures (CLP) regulation of the EU [11]. Building materials such as wood-based panel products and furniture are the most common sources of indoor formaldehyde emission [12].

Emissions of formaldehyde from the wood-based panels can be reduced by several methods during the manufacturing process or by post-treatment of the wood-based panels. The most often used methods are: (a) Lowering the molar ratio of formaldehyde to urea in the UF resin; (b) the addition of formaldehyde scavengers of other compounds to the UF resin; and (c) the post-treatment of the wood-based panels with barrier layers or formaldehyde scavengers [13,14,15]. Barrier layers resulting from a surface treatment method work as an emission barrier for formaldehyde and volatile organic compounds (paint, UV topcoat, vinyl resin system, phenolic saturated film, melamine saturated paper, foil resin system, powder coating, etc.) [16,17,18].

The adhesive formulation of wood-based panels consists of resin and filler. Fillers are used to increase control rheology, viscosity, and to reduce the costs of the raw materials. Filler is also used to supporting the bonding between the components. On the other hand, filler helps to reduce the penetration of resin into the small pores of the wood [19]. Many types of fillers, mostly natural, were investigated by other researchers, for example, corn starch flour, wheat flour, soybean meal, sorghum flour, and palm kernel meal [20,21,22,23,24,25,26]. In recent years, some research studies have been conducted to prepare and study the reaction mechanism and structure of environmentally friendly urea-based aminoresins by choosing glyoxal to substitute formaldehyde [27]. 

Using the tree bark to substitute technical flour as a filler is another way to reduce the negative environmental impact of the wood processing industry. Bark is basically a waste product of wood processing. Bark accounts for 6–9% of the dry mass of unprepared timber, depending on the type of hardwood and the log diameter [28]. Every year, a great quantity of waste bark is produced by wood processing. The North American wood industries generate more than 50 million tons of bark annually. In Germany, 2 million tons of bark is generated annually [29]. Wood processing companies in Slovakia generate approximately 7.5 × 10^5^ m^3^ of waste bark. The most common way of utilizing the bark (except mulching) is to burn it, although the heat produced by bark is lower (only about 60% of wood heat) and has a high ash content of about 3.5%. Therefore, bark is less suitable for energy production [30]. The replacement of technical flours in adhesive mixtures is also of great importance for creating space for the greater use of food flour in the food industry, thus eliminating the problem of threatening the food security of the population [31].

Using bark in EWPs has been investigated over last few years. Pedieu et al. [32] showed that 70% of wood fibers in the core layer of particleboard could be replaced by white birch inner bark while maintaining the required mechanical and physical properties. Another study by Cetin et al. [33] showed that bark in the core layer significantly lowered the formaldehyde emissions of the particleboard. The positive effect of amount of bark used to replace technical flour was observed only in the case of specific concentrations. The formaldehyde release, thickness swelling, and mechanical strength worsened significantly when the amount of bark was higher than 12.25% [34]. Following the study, the physical and mechanical properties required for furniture manufacturing were sufficient while the bark content was under 30% [35]. The area of bio-based adhesives on the basis of bark liquefaction for particleboard bonding is also interesting [36]. The quality of the final composite material is affected by the pressing process as well. Temperature, pressure and time are the most important factors of the pressing process [37,38,39]. Key aspects also include the adhesive properties, mainly the temperature and the hardening time [40]. A significant effect of adhesives on heat transfer in the pressing process was described in some research studies, for example in the study by Demirkir et al. [41].

This study follows our previous research [37]. In this study, the effect of various compositions of adhesive mixtures containing beech bark as the filler on the pressing process, physical and mechanical properties, and formaldehyde emissions of plywood were investigated in more detail. Besides the use of a reference adhesive mixture (UF adhesive, technical flour as a filler and hardener), three new, more preferable and different adhesive mixtures containing beech bark were used. 

The complex implication of the behavior of the beech bark-based filler in UF adhesive mixtures and its impact on the improvement/degradation of some properties of the produced plywood is the benefit of this research. It is important to note that the beech bark-based filler can be characterized as an environmentally friendly alternative. It is a renewable source of raw material, and therefore its increased use will make more food flour available for nutrition and feeding.

## 2. Materials and Methods

Under laboratory conditions, test groups of 5-ply plywood, made from beech (*Fagus sylvatica* L.) veneers, were glued together with adhesive mixtures of different composition. Beech from the central region of the Polana Mountains in Slovakia was used. Beech veneers were made via the centric peeling process using a 4-foot lathe (Královopolská strojírna, Brno, Czech Republic) at the Technical University in Zvolen, Slovakia. The average thickness of veneers was 1.23 mm. The veneers with the dimensions of 480 mm × 480 mm were cut. Their moisture content after drying and conditioning was 5–7%. 

UF adhesive Kronores CB 1100 F (DIAKOL Strazske s.r.o., Strážske, Slovakia), with a dry matter content of 67%, a viscosity of 1000–2000 mPa·s, a condensation time of 55 s, and a pH value of 8.5–8.8 was used to bond the veneers. The ammonium nitrate hardener NH_4_NO_3_ (47%) (DIAKOL Strazske s.r.o., Strážske, Slovakia) was used for curing. The hardener was added at a ratio of 10 g per 100 g of adhesive. This effective and reactive hardener was used in order to shorten the pressing time to a minimum, to provide maximal free formaldehyde binding and neutrality to the grinded bark to be used as the filler [42].

In the study, the grinded beech bark was selected as the filler of UF adhesive compositions for the production of plywood. After the drying process, the beech bark was grinded and then sieved (mesh number 60). Only the finest fraction of bark with grains smaller than 0.25 mm was added to the adhesive mixture. The size of grains in the fraction was almost identical to that of the technical flour. The moisture content of the bark was the same as the moisture content of the flour in order to not to affect the pressing conditions. 

Adhesive mixtures using bark were prepared. Firstly, bark was gradually added to the urea-formaldehyde resin to obtain a homogenous glue adhesive. Then, the hardener was added. The adhesive compositions used are shown in Table 1. Four compositions were formed: Reference one and three compositions using bark as the filler, K10, K15 and K20 (10 g, 15 g and 20 g of tree bark).

The adhesive mixtures were applied to the veneers with a hand roller to form the most uniform adhesive layer. The adhesive layer deposit of the veneer, with dimensions of 480 mm × 480 mm, was calculated following the basic adhesive layer deposit for urea formaldehyde adhesive mixtures per 1 m^2^ (180 g/m^2^). When composing the veneers, the fibers of the neighboring veneers were at a 90° angle, in accordance with the standard EN 636:2012 [43]. 

The pressing process was carried out using a single opening laboratory press (CBJ250, TOS Rakovník, Rakovník, Czech Republic). The pressing temperature was 105 °C (following the recommendation of the adhesive producer) and the pressure was calculated to be 9.6 MPa, due to the veneer sheet, tree species, and piston diameter of the pressing machine. The pressing time was 324 s and it was calculated as the sum of the basic pressing time for UF adhesives and the corresponding thickness of the pressed veneers. 

The plywood, after pressing, was conditioned at 20 ± 2 °C with a 60–70% relative moisture content for 4 weeks. The moisture contents after conditioning were calculated according to the standard ISO 13061-1:2014 [44]. After conditioning, the plywood was cut into test samples according to the standard EN 326-1 [45].

### 2.1. Heat Transfer in Pressing Process

**Figure 1:**
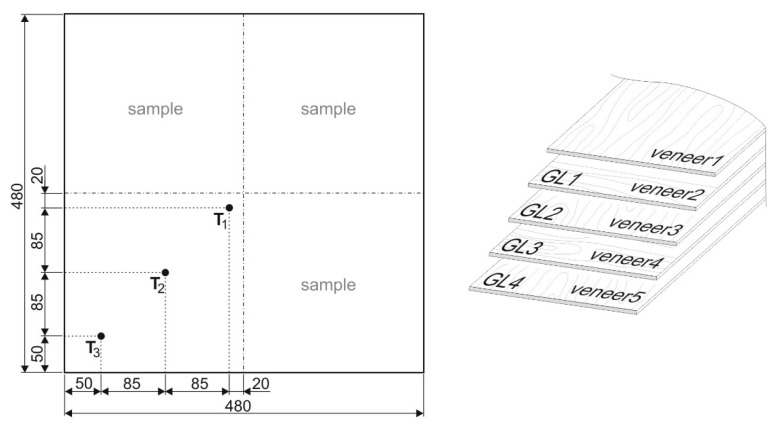
Composing veneers with adhesive mixture applied with fixed sensor to monitor the temperature flow inside the board during the plywood pressing process.

A temperature of 105 °C in the adhesive layer furthest from the surface of the pressed plywood must be achieved for the appropriate curing of the UF adhesive. For this purpose, the time taken to heat to 105 °C was evaluated. The composition of the veneer test groups was adapted as well: The temperature sensors were inserted between the third and the fourth veneer to monitor the temperature during the pressing process. Temperature measurement was carried out using three “K” thermocouples (Figure 1). Data from thermocouples were evaluated using the multimeter GW Instek GDM-8255A (Good Will Instrument Co., New Taipei City, Taiwan). 

The thermal conductivity of the beech bark and technical flour was also measured to find out what the effect of the replacement of technical flour by beech bark on the thermal conductivity of adhesive mixture was. This was done using two methods suitable for measuring the thermal conductivity of powders: A plane source method and hot wire method.

### 2.2. Viscosity

Viscosity is one of the most important parameters of adhesive mixtures. Adhesives with high viscosity are difficult to apply to the surface when the veneer surface has not been wetted enough. On the other hand, adhesives with low viscosity penetrate into veneers and thus a poor bond strength is produced. Dynamic viscosity was measured using a rotary viscometer (Cannon Instrument Company, State College, PA, USA), following the standard test method (ASTM D1084-16) [46]. 

### 2.3. Digital Microscopy

The detailed structures of liquid adhesives before the curing process and the uniform distribution of fine particles of grinded bark within the liquid environment was evaluated using the digital microscope Keyence VHX-5000 (Keyence, Mechelen, Belgium). This type of optical microscope, with a large depth-of-field and advanced measurement capabilities, was able to inspect the detailed structure of liquid adhesives.

### 2.4. Mechanical Properties 

The bending strength and modulus of elasticity of the plywood panels was determined according to the standard EN 310 [47]. The perpendicular tensile strength was tested according to EN 319 [48]. 

### 2.5. Formaldehyde Emissions

In the case of formaldehyde emissions, the EN 717-2:1995 [49] method was used. A glass desiccator with distilled water was used for free formaldehyde emissions from the plywood within 24 hours. A SPEKOL 221 spectrophotometer (Carl Zeiss, Jena, Germany) was used to determine the total formaldehyde content.

## 3. Results and Discussion

### 3.1. Heat Transfer in the Pressing Process

**Figure 2:**
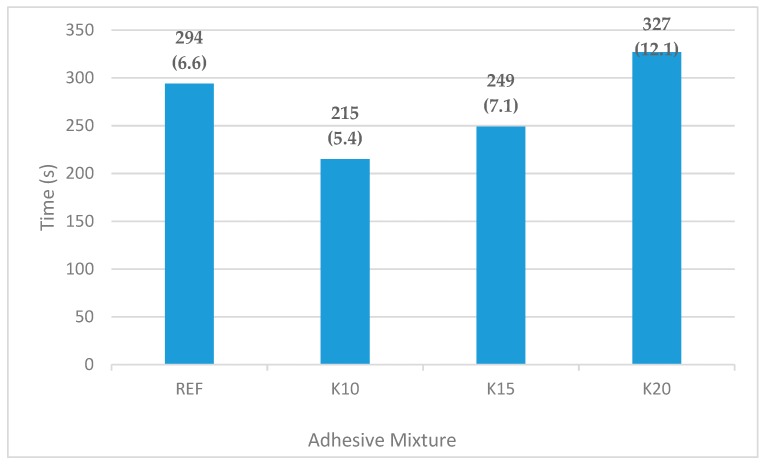
Time taken to heat to 105 °C in the centre of the adhesive layer (values in parenthesis are standard deviations).

The effect of the adhesive mixture formulation on the time necessary to heat up the test groups during pressing was studied in the experiment. The time necessary to heat up the sample to the required temperature (105 °C) in the centre of the adhesive layer was observed. The average values of the time measured in the case of tested adhesive mixtures REF, K10, K15, K20 are shown in Figure 2. 

An increase in the bark/adhesive ratio results in higher water binding in adhesive mixture. This way, water is bonded with bark (due to the large inner and outer surface of the bark). Therefore, less water penetrates into the veneers, and their thermal conductivity does not increase. As a result, the time necessary to heat the adhesive layer to specified temperature is longer. On the contrary, in the case of a decrease in the bark/adhesive ratio, a higher amount of water remains in the adhesive mixture and is absorbed into the wood. Subsequently, the moisture content increases, the thermal conductivity increases as well, and the heat transfer in the pressing process is quicker. When the bark/adhesive ratio decreases, the time taken to heat it up shortens (see samples K10 and K15). The results are in compliance with Ong et al. [22]. 

In addition to the time taken to heat up the adhesive layer to the specified temperature, the coefficient of thermal conductivity (*λ*) of the technical flour and bark was measured using the plane source method (PS) and hot wire method (HW) [50]. 

The value of the coefficient of thermal conductivity of the technical flour was statistically significantly higher than that of the beech bark (Table 2). When the concentration of technical flour (REF) and bark (K20) was identical, the time needed to heat up the adhesive layer to the specified temperature was shorter in the case of the reference sample. The addition of 20 units of grinded bark (a replacement of 20 units of technical flour) results in worse heat transfer in the pressing process of the plywood and thus in an increase in the time necessary to reach a temperature of 105 °C. 

### 3.2. Viscosity

**Figure 3:**
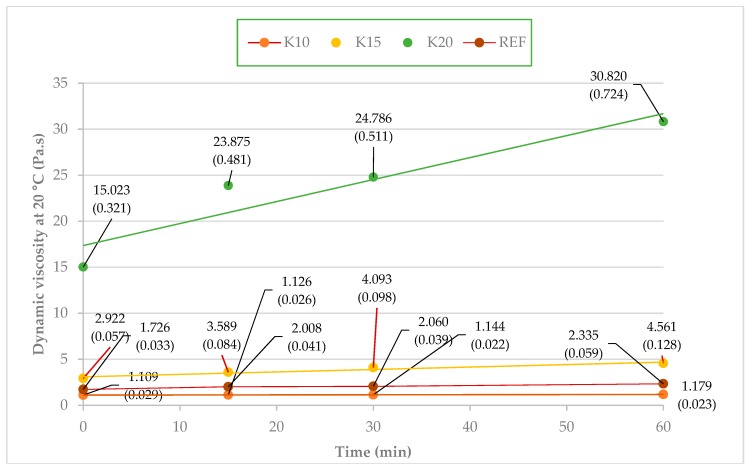
Time-viscosity dependence at 20 °C for the adhesive mixtures REF, K10, K15, and K20 (values in parenthesis are standard deviations).

The dependence of the dynamic viscosity of adhesive mixtures over time was analyzed in the study. The time-viscosity dependence is illustrated in Figure 3. The method of least squares was used to evaluate the values of viscosity. The values of correlation corresponding with the adhesive mixtures REF, K10, K15, K20 are 0.972, 0.999, 0.967, and 0.940, respectively. The time-viscosity dependence can be considered to be linear as adhesive mixtures act like Newtonian fluids. Adhesive mixtures K10 and K15 can be applied easily on veneers. Adhesive mixture K20 is applied on veneers with difficulties due to its high viscosity, therefore it cannot be used in practice. 

Time instability in UF resins is caused by the progress of polycondensation reactions, resulting in an increase in resin viscosity. The dependence of dynamic viscosity of the adhesive mixtures over time was acceptable in cases of the mixtures REF, K10 and K15. An increase in dynamic viscosity can be compared to other authors, e.g., Pereira et al. [51,52,53]. 

Grinded beech bark results in acceptable values of viscosity for adhesive mixtures with a lower concentration (K10, K15), as in case of technical flour. High viscosity, in the case of K20, is caused by a large quantity of fine dust particles, together with the large inner surface of the bark binding water. This explanation is also in accordance with the results gathered by measuring the time needed to reach the specified temperature in the adhesive layer (Section 3.1). In the case of too much bark in adhesive mixture, the bark bonds almost all the water from the adhesive mixture, which leads to very high viscosity. Another problem is that a large amount of bark, and subsequently tannin, contains polyflavonoids that react with formaldehyde, forming condensation products. Its high reactivity at high concentrations results in a quick increase in viscosity, short pot life of the resin, and subsequent lower crosslinking, corresponding with the results by Yazaki and Collins [54].

### 3.3. Digital Microscopy 

The use of the digital microscope in the case of the tested adhesive mixtures (REF, K10, K15, and K20) confirmed the fact that the fine particles of the grinded bark were distributed correctly in the adhesive mixtures. A good distribution of fine bark particles in adhesive mixtures is a prerequisite for achieving a quality bonded joint. The correct size of the small bark grains used was confirmed by the microscopic images.

**Figure 4:**
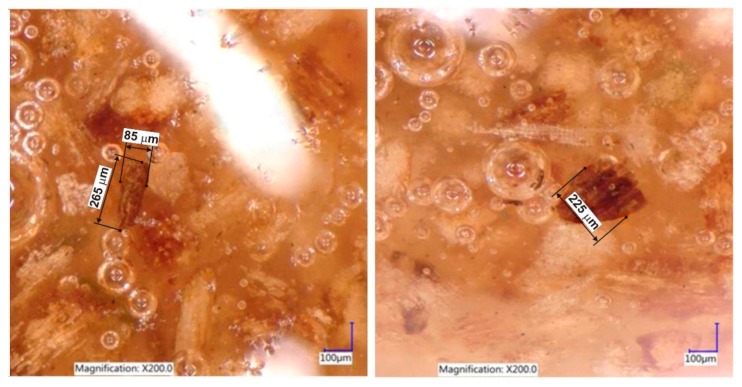
The particles of ground bark scattered in the adhesive mixture.

The bark particles scattered in the adhesive mixture are clearly illustrated in Figure 4. The adhesive mixture is homogeneous after stirring. Only reflections of the light rays from the microscope are visible, but they do not reduce the image quality. The bark element sizes indicate good and homogeneous laboratory grinding of the bark. The bark particles in the homogeneous adhesive mixture are dispersed evenly and the interaction with the adhesive mixture is adequate. Following the microscopic images, even after grinding, no agglomerations of bark particles could be seen. The bark particles in the adhesive mixture did not settle during one working shift (8 h). This was confirmed by the microscopic images, which were scanned gradually for 2–8 h. It is very important in terms of the stability and sufficient life of the bark mixture. This corresponds with the results of Kim et al. and Siimer et al. [55,56]. The bark particles did not react with the adhesive prematurely and they behaved in a completely neutral way. Following the microscopic images, we can state that the bark particles in the adhesive mixture are functional, that they perform the role of the filler in the adhesive composition, and that they create the necessary environment of the glue mixture for pressing the plywood.

**Figure 5:**
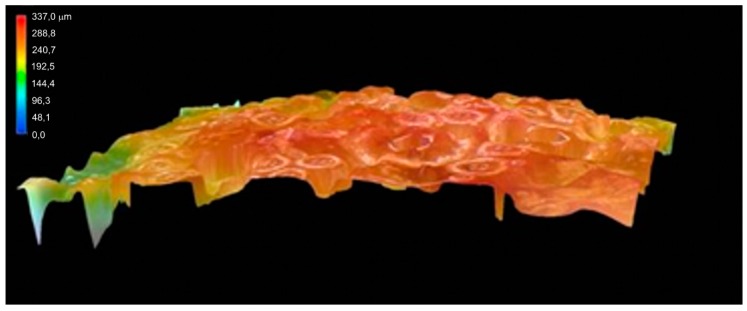
The particles of ground bark scattered in the adhesive mixture.

Figure 5 shows the 3D confocal microscopy of the “oversized droplet” surface of the adhesive mixture with bark particles. After sufficient mixing, they were dispersed throughout the total volume of the adhesive mixture. They do not crack out of the “droplet” surface and the “droplet” surface is continuous. The condition of uniform dispersion in the adhesive mixture is the correct adequate pulverization of the bark and the achievement of the uniform granularity. Bark particles with dimensions of up to 300 μm probably do not cause any difficulties in the adhesive mixture and such a size of evenly ground particles can be recommended as the corresponding size of the bark usable for the technological purposes of manufacturing plywood materials.

### 3.4. Mechanical Properties 

#### 3.4.1. Bending Strength and Modulus of Elasticity

**Figure 6:**
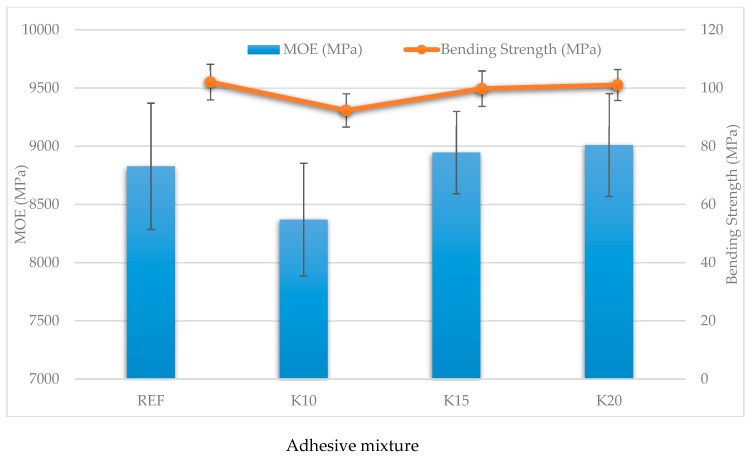
Effect of adhesive mixtures on bending strength and modulus of elasticity of plywood panels.

The experimentally made plywood was analyzed using the three points bending test. The quality of the bonded joint was tested using the bending test. Average values and deviations of the experimentally determined mechanical properties are mentioned in Figure 6.

The results of the tested modulus of elasticity and bending strength show that the grinded beech bark in the adhesive mixtures K10, K15 and K20 can be used to equally substitute the commonly used technical flour. The change in values was not statistically significant. The results correspond with the analysis in Section 3.1 and Section 3.2. A small increase in the bending strength and modulus of elasticity of the plywood panels can be seen when the quantity of added bark increases. This can be explained by the tannin content in the bark and its positive effect on the bending strength and modulus of elasticity of the plywood panels [34,57,58]. This statement does not work in all cases of tannin-based adhesives. Kim et al. [59] investigated the physical and mechanical properties of particleboard made using two types of tannin-based adhesives, wattle and pine, with three hardeners. The bending strength of the particleboard made using the wattle tannin-based adhesive with all three hardeners increased slightly, while in case of the pine tannin-based particleboard, a decrease in bending strength was observed. As the authors stated, wattle tannin-based adhesive is a thermoset influenced more by physical conditions, while the pine tannin-based particleboard was influenced by the chemical structure of the pine tannin nuclei, including phloroglucinolic A-rings with a reactivity over 50. These adhesives were cured early, resulting in brittle particleboard with lower values for the mechanical properties. Our results also correspond with results of Muszynski et al. [60], who found out that in the case of bark substitution, up to 30% in UF adhesive, the physical and mechanical properties of the particleboard could still meet the requirements for furniture manufacturing. The beech bark-based filler used in the adhesive composition does not impair either the adhesive composition or the final plywood product and therefore it is suitable for use in assembly.

#### 3.4.2. Perpendicular Tensile Strength 

**Figure 7:**
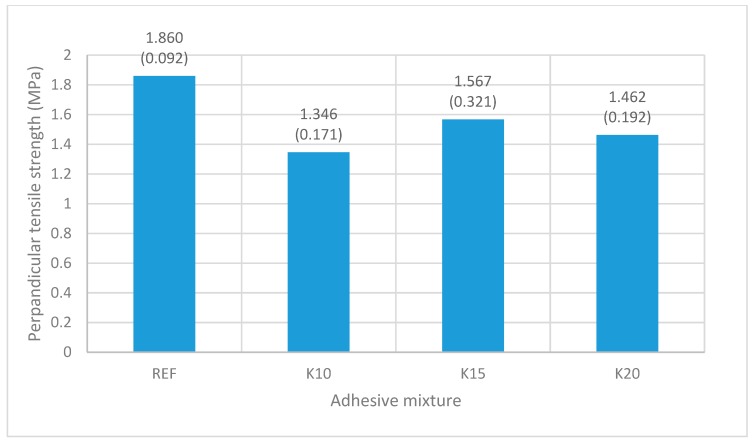
Effect of adhesive mixtures on perpendicular tensile strength of plywood panels (values in parenthesis are standard deviations).

Figure 7 shows the values for the perpendicular tensile strength of all of the tested plywoods with varying adhesive mixtures (REF, K10, K15, K20). All perpendicular tensile strength mean values obtained were above the limit (1 MPa) given in the standard EN 319. 

Comparison of plywood with the adhesive mixtures K10 and K15 shows a slight increase in perpendicular tensile strength, resulting from an increase in the quantity of bark in the adhesive mixture. This is due to the tannin in the bark, which improves the adhesion properties. The same results were observed by other authors [61,62]. However, a further increase in the bark/adhesive ratio (K20) results in a slight decrease in the perpendicular tensile strength. The first reason for this is a lack of water, as explained in the viscosity section. Water reacts with UF resin, containing the hydroxymethyl groups and isocyanate groups in the mixture and forming a three-dimensional crosslinking network structure which enhances the bonding strength. Another reason is due to the high viscosity of the adhesive mixture K20 and the high tannin/adhesive ratio in the adhesive mixture. Due to high reactivity at higher concentrations, a rapid increase in viscosity and a short pot life of resin is observed, resulting in lower crosslinking. This corresponds with the fact that the perpendicular tensile strength of the plywood panels made using tannin-based adhesives does not depend only on the adhesive mixture, but mainly on the physical conditions, such as pressure and press temperature, as well as by chemical conditions, which is confirmed by the research studies by Aydin et al. and Rhazi et al. [34,63]. Perpendicular tensile strength increases when the cure time and press temperature increase. More energy is needed for crosslinking the bark or tannin. As a result, molecules of the tannin prepolymer and functional groups are mutually crosslinked to each other. That is why the value of perpendicular tensile strength of reference sample is higher than in the case of the bark samples. Despite the lower values of perpendicular tensile strength for the samples with bark, the values remain within the standard EN 319. 

### 3.5. Free Formaldehyde Emissions

**Figure 8:**
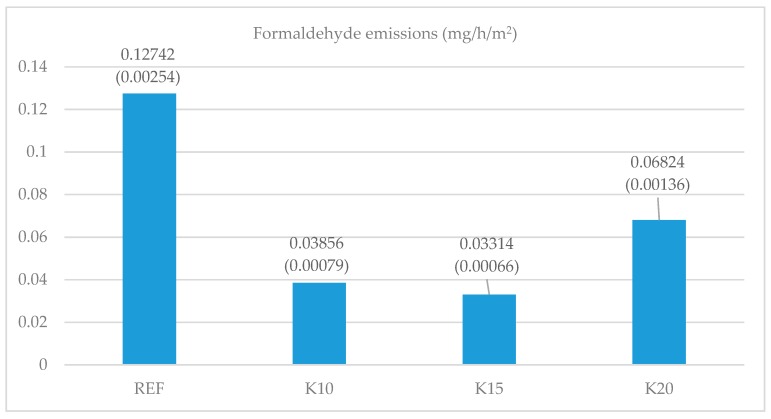
The effect of adhesive mixtures on formaldehyde emissions (values in parenthesis are standard deviations).

Free formaldehyde emissions in the samples are shown in Figure 8. The replacement of technical flour by beech bark led in all cases to a significant decrease in free formaldehyde emissions when compared to the reference sample. The formaldehyde emissions decreased from 46% to 74%.

Bark as the filler in the adhesive mixture leads to a significant decrease in free formaldehyde release in plywood production. This is due to the tannin in the bark, which is in a condensed polyflavonoid form with a phenolic nature, leading to a decrease in formaldehyde emissions, as was confirmed by some studies [64,65,66]. A further reduction of formaldehyde emissions, but only to a certain value, occurs because of the increase in the amount of bark and thus because of the increase in the share of tannin. In the case of too much bark in adhesive mixture, the bark bonds to almost all of the water from the adhesive mixture and cannot be absorbed into the wood veneers. This results in a significant decrease in moisture content in the veneers, which is known to increase formaldehyde emission. The solution of this problem can be solved by adding some water to the adhesive mixture. This problem is worth further research.

A significant decrease in formaldehyde emissions with the increased use of bark was also studied in particleboard manufacturing [67]. It was demonstrated that the formaldehyde release deteriorated significantly at bark addition levels from 12.25% to 25.00%. A decrease in formaldehyde release was also confirmed in research into thermal insulation using bark [68].

## 4. Conclusions

The objective of the research was to evaluate the utilization of grinded beech bark (*Fagus sylvatica* L.) as the filler in adhesive mixtures designated for plywood production. The application of the replacement of technical flour was evaluated in terms of various aspects of the technological and functional properties. Plywood made using a standard formulation of adhesive mixture under identical conditions of pressing was used as a reference material.

Under laboratory conditions, test groups of 5-ply plywood were made of beech veneer and bonded together with three alternatives of adhesive mixtures with different beech bark-based fillers. The present study was focused on evaluating the utilization of beech bark as an environmentally friendly filler in UF adhesives. 

Following the results, the following findings can be stated:

- The replacement of technical flour with beech bark (with an identical adhesive mixture, 20 g) results in a slight decrease in heat transfer during the pressing process. Subsequently, longer time is taken to reach the specified temperature in the central adhesive layer (due to the lower thermal conductivity of the bark).
- The strength of the bonded joints is significantly affected by the viscosity of adhesive composition. In terms of technological properties, the most suitable beech bark/adhesive ratio ranged from 0.10 to 0.15. Too high of a viscosity of the adhesive composition K20 makes it impossible to apply the adhesive mixture to the veneer surfaces by standard methods.
- Technical flour in the adhesive mixture (20 g) can be replaced with grinded beech bark (10–20 g) with no statistically significant change in the modulus of elasticity and bending strength of the plywood. In the case of a bark/adhesive ratio of 0.15 and 0.20, a slight increase in observed properties was observed.
- Microscopic details point out the realistic dimensions of beech bark-based filler proportionally dispersed in the adhesive mixture. They also show that the beech bark-based filler is sufficiently dispersed in the liquid adhesive composition and that there are no disproportionate clumps.
- The values of perpendicular tensile strength in all bark/adhesive ratios as the filler meet the requirements of the applicable standards.
- Utilization of the adhesive mixtures with the beech bark-based filler led to decrease in free formaldehyde release compared to the reference sample. The formaldehyde release decreased to about 75%, which can be considered to be a significant contribution in terms of the environment.
- The performance of the plywood panels is influenced by physical conditions such as pressure and press temperature, as well as by chemical conditions. These results provide ideas for further research into different pressing parameters.
- Utilization of waste products in wood processing results in another benefit of using beech bark as the filler. Bark is typically considered to be a waste, where it is used as a low-quality fuel or it is mulched.

Following the results of the research, grinded beech bark can be characterized as an environmentally friendly alternative to technical flour in UF adhesive mixtures designated for plywood production. In terms of the technological and functional properties, plywood made using the adhesive mixtures with beech bark-based filler is an alternative to plywood made using standard methods with standard adhesives. In addition, further possibilities to optimize the technological parameters of plywood production, especially the pressing process, can be studied in more detail. Looking for ways to utilize waste products industrially is in compliance with the concept of utilizing waste products, reducing the environmental impact and long-term sustainability of industrial production, resulting in a decrease in the environmental impact of waste generated during wood processing [69,70]. 

## Figures and Tables

**Table 1:** Composition of tested adhesive mixtures.

Adhesive Mixture	REF	K10	K15	K20
**Urea Formaldehyde (UF) Adhesive**	100 g	100 g	100 g	100 g
**Filler**	20 g technical flour	10 g beech bark	15 g beech bark	20 g beech bark
**NH_4_NO_3_ Hardener**	10 g	10 g	10 g	10 g

**Table 2:** Thermal conductivity of technical flour and bark determined using the plane source method (PS) and hot wire (HW) method (values in parenthesis are standard deviations).

Technical Flour λ (W·m^−1^·K^−1^)		Beech Bark λ (W·m^−1^·K^−1^)	
Plane source (PS) method	Hot wire (HW) method	PS method	HW method
0.131 (0.002)	0.126 (0.002)	0.105 (0.001)	0.102 (0.001)
